# Interobserver variation in rectal and bladder doses in orthogonal film-based treatment planning of cancer of the uterine cervix

**DOI:** 10.4103/0971-6203.44476

**Published:** 2008

**Authors:** P. Raghukumar, K. Raghu Ram Nair, B. Saju, G. Zhenia, K.T. Divya, V.S. Shaiju, V. Padmanabhan

**Affiliations:** Radiation Physics Division, Regional Cancer Centre, Trivandrum, Kerala-695 011, India; 1 SUT Hospital, Trivandrum, Kerala-695 005, India

**Keywords:** High dose rate brachytherapy, interobserver variation, uterine cervix

## Abstract

Orthogonal film-based treatment planning is the most commonly adopted standard practice of treatment planning for cancer of the uterine cervix using high dose rate brachytherapy (HDR). This study aims at examining the variation in rectal and bladder doses when the same set of orthogonal films was given to different observers. Five physicists were given 35 pairs of orthogonal films obtained from patients who had undergone HDR brachytherapy. They were given the same instructions and asked to plan the case assuming the tumor was centrally placed, using the treatment-planning system, PLATO BPS V13.2. A statistically significant difference was observed in the average rectal (F = 3.407, *P* = 0.01) and bladder (F = 3.284, *P* = 0.013) doses and the volumes enclosed by the 100% isodose curve (*P* < 0.01) obtained by each observer. These variations may be attributed to the differences in the reconstruction of applicators, the selection of source positions in ovoids and the intrauterine (IU) tube, and the differences in the selection of points especially for the rectum, from lateral radiographs. These variations in planning seen within a department can be avoided if a particular source pattern is followed in the intrauterine tube, unless a specific situation demands a change. Variations in the selection of rectal points can be ruled out if the posterior vaginal surface is clearly seen.

## Introduction

Cancer of the uterine cervix has a high incidence rate among women in India. Most of the radical cases are treated by combining external beam therapy and brachytherapy.[1–2] Low-dose rate (LDR) brachytherapy has radiobiologically proven its role in controlling the tumor with acceptable late morbidity. [3–8] HDR overcomes the disadvantages of LDR brachytherapy with the added advantages of reduced treatment times and flexibility in dose optimization. Although the dose is delivered at a higher rate than in LDR, the possible late effects can be reduced by adopting low fraction size and multiple fractions with adequate gap in between fractions.[3,6,7] All fractions require careful individualized planning due to the geometrical variation of applicators arising from the differences in the anatomy of the patient and variations in packing etc for keeping the rectal and bladder doses within acceptable limits. [9–17] Orthogonal film-based planning is the standard practice recommended by the American Brachytherapy Society[18] and this is being followed in most of the centers in India. The reconstruction of catheters from X-ray markers, the selection of ICRU rectal and bladder points,[19] and hence, the selection of dwell positions are purely dependent on the physicist and the clinician associated with the procedure. This study aims at finding the variations in rectal and bladder doses and the dimensions of the 100% isodose volume when the same set of orthogonal films are used for planning by different observers.

## Materials and Methods

**Figure 1:**
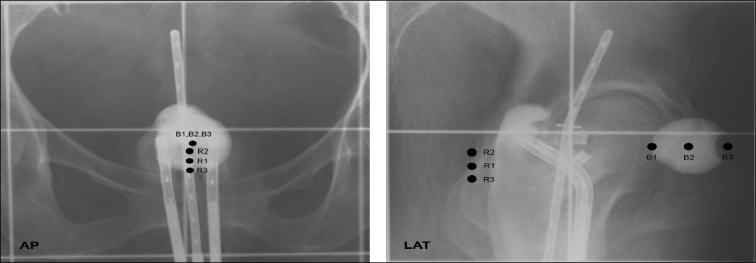
Selection of Rectal and Bladder Points in AP and LAT radiographs

The Regional Cancer Centre, Trivandrum treats nearly 35 patients per week using a Microselectron HDR brachytherapy machine. All patients are simulated and after confirming the adequacy of the applicator position, two orthogonal films are taken with a magnification of 1.5. Thirty-five pairs of orthogonal films were selected randomly for this study from the records for which planning had already been done and treatment executed. A general guideline was given to the planners as to the magnification of the films, the beam direction (AP and Right to left), and how to keep the films without lateral inversion. All the five observers were requested to carry out planning independently, assuming that the tumor was centrally placed. They were asked to select rectal and bladder points as per ICRU recommendations [Figure 1] and normalize the dose to point A. Treatment planning was done using PLATO BPS V.13.2. by feeding data from the films to the system via a digitizer. The data obtained through the plan was not used for treatment purposes, but was only considered for studying the interobserver variations in planning.

## Results and Discussion

The rectal and bladder doses obtained after planning were noted from the final plans of each observer. Three points were selected for the bladder: the first point was the posterior-most point of the Foleys balloon, closer to the applicator where the maximal dose is obtained (B1), the second point was in the center (B2), and the third point (B3) was at the other end of the balloon. B1 is the ICRU bladder point; B2 and B3 were selected to assess the dose fall-off. The observers were also asked to select three rectal points: one the ICRU rectal point (R2), the second 0.75 cm above R2 (R1), and the other 0.75 cm below R2 (R3) on the film. Statistical analysis (ANOVA) was done on the average rectal (R_avrg_) and maximal bladder doses (B1) calculated by the five observers. Significant differences in rectal (F = 3.407, *P* = 0.01) and bladder (F = 3.284, *P* = 0.013) doses were observed between the observers.

As all were given the same film, the geometry of the applicator and rectal and bladder packing could be assumed to be the same for all. Clear specifications were given regarding the orientation of the film on the digitizer and the orientation of the beam for taking the lateral film. This resulted in no significant difference in the reconstruction of the ovoid sources, unless there was difficulty in distinguishing the first and second catheters due to overlapping of the ovoids from lateral films. Hence, the variations in the rectal doses may be partly due to the selection of the rectal points from the radiographs and the selection of dwell positions in the catheters. In some cases, it was difficult for the planners to find the vaginal surface from which the ICRU rectal point should be marked. Hence, the selection of rectal points from such radiographs was purely based on their experience.

The average percentage of rectal and bladder doses obtained by each physicist is given in Table 1; a break-up of the percentages of the doses obtained for each group is also provided. From the table, it is clear that when one tries to reduce the dose to one organ, the dose to the other organ is found to increase. Physicist C could plan 57% of the cases with a minimum average dose of 80–100% to the bladder, but only 43% of the cases received a dose of ≤ 70% to the rectum. Physicist B tried to keep the rectal percentage ≤ 70% for 57% of the cases planned. Physicist A could limit the dose to the rectum in 50% of the cases, but the number of patients receiving doses > 100% in the bladder was comparatively higher (60%) in A's case. Physicist D limited the dose to the minimum level in 26% of the cases.

**Table 1:** Percentage of patients receiving a given bladder and rectal dose (%) of Point A dose in the plans done by the five physicists

*Percentage of patients*	*Physicist A*	*PhysicistB*	*PhysicistC*	*PhysicistD*	*PhysicistE*
Bladder (%)					
≤ 80	6	11	14	26	14
80–100	34	46	57	49	43
> 100	60	43	29	25	43
Rectum (%)					
≤ 70	50	57	43	26	34
70–80	17	20	26	26	15
80–100	28	20	28	39	31
> 100	5	3	3	9	20
Mean (%) Bladder dose	104.4	98.3	72.9	80.9	83.3
Mean (%) Rectal dose	74	71.4	72.9	80.9	83.3

The dwell positions selected by each observer in each ovoid and in the intrauterine tube are given in Table 2. In most of the cases, all observers selected the same number of dwell positions (three) in each ovoid. However, the selection of the dwell positions in the intrauterine tube differed among the physicists [Table 3]. The selection of dwell weightage, which is the ratio between the number of sources in the IU and the total number of sources in the ovoids, was in the ratio of 1:1.2 for physicists A and B in almost all the cases they planned whereas physicist C had a dwell weightage mixture of 1:1.2 and 1:1. Physicist E had a ratio of 1:1 in 95% of the cases whereas physicist D adopted a ratio of 1.2:1 for planning the cases.

**Table 2:** Source dwell positions selected by each physicist for the ovoids and IU tube

*No. of Dwell Positions*	*Number of cases*				
	*PhysicistA*	*PhysicistB*	*PhysicistC*	*Physicist D*	*PhysicistE*
	
Each Ovoid					
2	1	5	6	0	0
3	32	30	29	32	33
4	2	0	0	3	2
IU Tube					
5	35	30	15	0	2
6	0	5	18	7	32
7	0	0	2	28	1

**Table 3:** Parameters of 100% isodose volume

Dimension in cm	Physicists				
	A	B	C	D	E
Dw	5.7	5.5	5.5	5.3	5.4
Dh	8.3	7.7	7.8	7.9	8.1
Dt	4.6	4.3	4.1	4.1	4.2
Volume (cm^3^)	219.6	185.4	185.1	172.5	181.7

**Figure 2:**
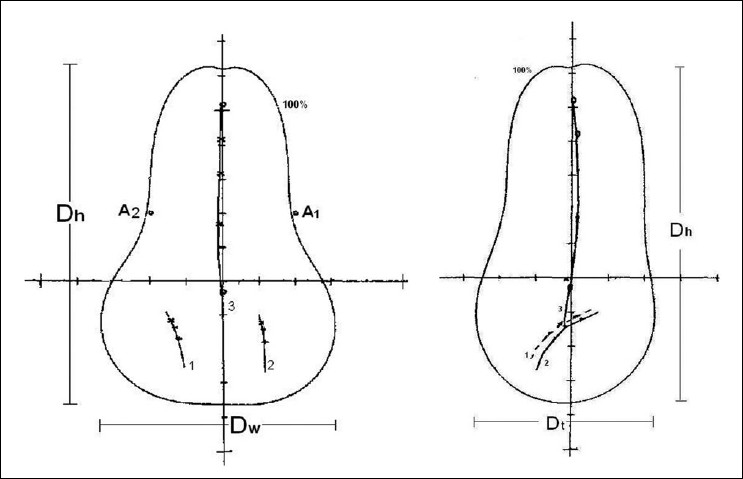
Diagram showing source positions in the applicator, 100% isodose volume passing through A1, A2 and the height (Dh), width (Dw) and thickness (Dt) of the isodose volume

A study of the width (D_w_), height (D_h_), thickness (D_t_) [Figure 2] and volume encompassed by the 100% isodose curve revealed that a significant variation existed in all the parameters (*P* < 0.01) between planners. Table 3 shows the average values of D_w_, D_h_, D_t_ and the volumes (cm^3^) obtained by different observers.

## Conclusion

There will be variations in rectal and bladder doses when the applicator placement is done by different observers.[20] The same plan followed by different individuals in a department will bring up differences in rectal and bladder doses with each observer. This can be avoided if all physicists within the department follow the same source pattern and number of sources, unless a specific situation arises to adjust the dwell positions in the intrauterine tube. The source selection in the ovoids is mainly dependent on the shift of ovoids in the anterior-posterior direction, which cannot be controlled. However, the number of source positions in the ovoids can be made constant. The flexibility of using any position can be utilized as and when the need arises during planning. The dose variations in the rectum can be brought to the minimal limit if the selection of the rectal point is done according to ICRU-38 guidelines. The selection of the rectal point can be done more accurately if the posterior vaginal surface is clearly visible on the radiograph. Some centers use a radioopaque wire- threaded gauze piece for rectal and bladder packing. Some other centers use a barium-soaked gauze pack. In the latter, if the amount of barium is high, it will mask the ovoid catheters making the reconstruction of the applicator difficult. Use of flexible wire rectal markers, rectal balloons, and contrast media in rectum etc are other options available for rectal point identification. Currently, 3D image-based treatment planning is recommended as a more precise and accurate method of dose estimation in brachytherapy.[21]

This study shows that a standard protocol covering all the parameters mentioned above should be followed by all institutions wishing to implement HDR brachytherapy of cancer of the uterine cervix.
